# Bacterial endotoxin decreased histone H3 acetylation of bovine mammary epithelial cells and the adverse effect was suppressed by sodium butyrate

**DOI:** 10.1186/s12917-019-2007-5

**Published:** 2019-07-29

**Authors:** Jingbo Chen, Yongjiang Wu, Yawang Sun, Xianwen Dong, Zili Wang, Zhu Zhang, Yanli Xiao, Guozhong Dong

**Affiliations:** 1 College of Animal Science and Technology, Southwest University, Beibei, 400716 China; 2 Institute for Herbivorous Livestock Research, Chongqing Academy of Animal Science, Chongqing, 402460 China; 3 College of International Studies, Southwest University, Beibei, 400716 China

**Keywords:** Endotoxin, Lipopolysaccharide, Histone acetylation, Sodium butyrate, Bovine, Mammary, Epithelial cells

## Abstract

**Background:**

In practical production, dairy cows are frequently exposed to bacterial endotoxin (lipopolysaccharide, LPS) when they are subjected to high-concentrate diets, poor hygienic environments, as well as mastitis and metritis. Histone acetylation is an important epigenetic control of DNA transcription and a higher histone acetylation is associated with facilitated transcription. LPS might reduce histone acetylation in the mammary epithelial cells, resulting in lower transcription and mRNA expression of lactation-related genes. This study was conducted to investigate the effect of LPS on histone acetylation in bovine mammary epithelial cells and the efficacy of sodium butyrate (SB) in suppressing the endotoxin-induced adverse effect. Firstly, the bovine mammary epithelial cell line MAC-T cells were treated for 48 h with LPS at different doses of 0, 1, 10, 100, and 1000 endotoxin units (EU)/mL (1 EU = 0.1 ng), and the acetylation levels of histones H3 and H4 as well as the histone deacetylase (HDAC) activity were measured. Secondly, the MAC-T cells were treated for 48 h as follows: control, LPS (100 EU/mL), and LPS (100 EU/mL) plus SB (10 mmol/L), and the acetylation levels of histones H3 and H4 as well as milk gene mRNA expressions were determined.

**Results:**

The results showed that HDAC activity increased linearly with increasing LPS doses (*P* < 0.01). The histone H3 acetylation levels were significantly reduced by LPS, while the histone H4 acetylation levels were not affected by LPS (*P* > 0.05). Sodium butyrate, an inhibitor of HDAC, effectively suppressed the endotoxin-induced decline of histone H3 acetylation (*P* < 0.05). As a result, SB significantly enhanced the mRNA expression of lactation-related genes (*P* < 0.05).

**Conclusions:**

The results suggest one of the adverse effects of LPS on the lactation of bovine mammary gland epithelial cells was due to decreasing histone H3 acetylation through increasing HDAC activity, whereas the endotoxin-induced adverse effects were effectively suppressed by SB.

**Electronic supplementary material:**

The online version of this article (10.1186/s12917-019-2007-5) contains supplementary material, which is available to authorized users.

## Background

In practical dairy production, dairy cows are frequently fed high-concentrate diets to satisfy the high energy demand for milk production. High-concentrate diets often lead to increased yields of short-chain fatty acids (SCFAs) and lactic acid in the rumen, resulting in a decrease in rumen pH [1–3]. If rumen pH values remain below 5.6 for more than 3 h/day, subacute ruminal acidosis (SARA) occurs [4]. During high-concentrate feeding, particularly in the case of SARA, an increased amount of bacterial endotoxin (lipopolysaccharide, LPS) is released in the rumen fluid [1–3, 5]. Studies have shown that LPS in the digestive tract and blood increases significantly with increasing dietary concentrate ratios [5–8]. Our previous study also showed dairy cows fed high-concentrate diets had significantly higher LPS concentrations in the mammary arterial blood, as compared with cows fed the low-concentrate diets [9]. In practical production, dairy cows may also be exposed to LPS when they are subjected to poor hygienic environments. Furthermore, mastitis and metritis are common diseases for dairy cows, and under these conditions, LPS in the blood circulation and the mammary gland increases tremendously [3].

Once LPS enters the blood circulation, it can elicit systemic inflammatory response and local inflammatory response in the mammary gland [1]. Under the circumstances, more nutrients are used for synthesizing immune molecules, resulting in lower supply of substrate precursors for milk component synthesis and low milk production [1, 5, 10]. The entry of LPS into the mammary gland can also lead to production of an increasing amount of reactive oxygen species and NO, affecting the proliferation of mammary epithelial cells and even resulting in apoptosis of mammary epithelial cells [1, 5, 10]. LPS may also down-regulate the expression of milk genes such as fatty acid synthase (*FASN*) and acetyl-CoA carboxylase 1 (*ACACA*), resulting in lower milk yield [11, 12], probably through epigenetic manipulation.

Epigenetics is a science that studies heritable changes in gene expression that are independent of DNA sequence [13]. Major epigenetic events include histone modification, DNA methylation, and microRNA regulation. As far as histone modification is concerned, various histone modifications include acetylation, methylation, phosphorylation, and ubiquitinated succinylation, etc. [14, 15]. Histone is the basic structural protein of eukaryotic chromatin and binds most closely to DNA. Mammalian histones have five components, including H1, H2A, H2B, H3 and H4. The N-terminal of histones, especially histones H3 and H4, is very active because it extends out of the nucleosome. Therefore, histones are often chemically modified. The lysine residues in the N-terminal tail of histones are extremely active and can undergo chemical modifications such as acetylation. Acetylated histones can neutralize the positive charge of lysine residues, which reduces the binding of histones to DNA, making DNA adopt a more relaxed structure and thus facilitating transcription [16]. At present, the acetylation of histones H3 and H4 is the most frequently studied histone modification.

Histone acetyltransferase (HAT) and histone deacetylase (HDAC) are reciprocal enzymes involved in the histone acetylation process. Under the action of HAT, the acetylation levels of histones increases, whereas HDAC deacetylates histones, resulting in a closed state of chromatin and DNA as well as gene silencing [17]. Histone deacetylase inhibitors can inhibit deacetylation by HDAC. At present, the most well-studied HDAC inhibitors include sodium butyrate (SB) [18, 19], sodium valproate [20], and trichostatin [21], and SB is of great importance for ruminant animals [22, 23].

Up to now, it remains unclear whether LPS can induce hypoacetylation of histones H3 and H4 in bovine mammary epithelial cells and in which manner LPS can induce such changes, although our previous study showed that there existed a negative relationship between LPS concentrations in the mammary arterial blood and the histone H3 acetylation level in the mammary tissue of dairy cows [9]. Therefore, we hypothesized that LPS might decrease histone acetylation through increasing HDAC activity, and SB could effectively inhibit the HDAC activity, thus antagonizing the adverse effects of LPS on histone H3 and H4 acetylation. The objective of this study was to explore the effect of LPS on the acetylation levels of histones H3 and H4 in the bovine mammary epithelial cells and the possible enzymatic mechanism. Furthermore, we also investigate the efficacy of SB in reducing LPS-induced hypoacetylation of histones H3 and H4, so as to provide an insight into possible avenues to improve lactation performance in dairy cows.

## Results

### Effect of LPS on acetylation of histones H3 and H4

**Fig. 1:**
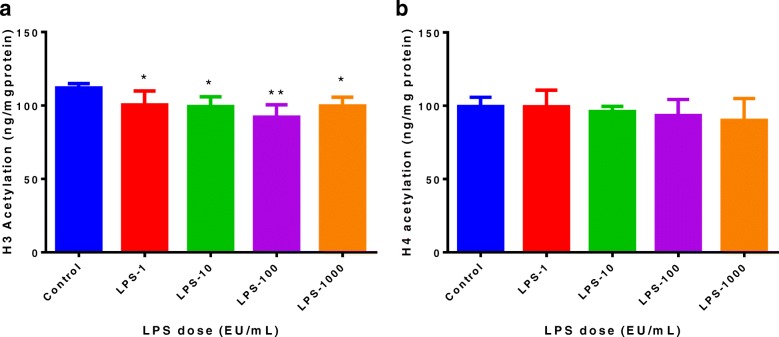
Effect of lipopolysaccharide (LPS) on histone H3 and H4 acetylation levels in MAC-T bovine mammary epithelial cells. The MAC-T bovine mammary epithelial cells were treated for 48 h with LPS at 0 (control), 1, 10, 100, 1000 endotoxin units (EU)/mL, respectively. (**a**) Histone H3 levels deceased significantly after LPS treatments (*n* = 5/treatment); (**b**) Histone H4 levels were not significantly (*P* > 0.05) affected by LPS treatments (*n* = 6/treatment). Data represent the mean and standard deviation and the asterisk indicates statistical difference between the indicated column and the control column (*, *P* < 0.05; **, *P* < 0.01). The raw data were shown in Additional file 1: Table S1

We are interested in determining the impact of LPS on the epigenetic changes in MAC-T bovine mammary epithelial cells. The results showed that the acetylation level of histone H3 decreased significantly after LPS treatment at different doses (Fig. 1a). The effect of LPS on histone H3 acetylation was not dose-dependent. LPS treatment tended to reduce the histone H4 acetylation level, but the effect was not statistically significant (*P* > 0.05) (Fig. 1b).

### Effect of LPS on HDAC activity

**Fig. 2:**
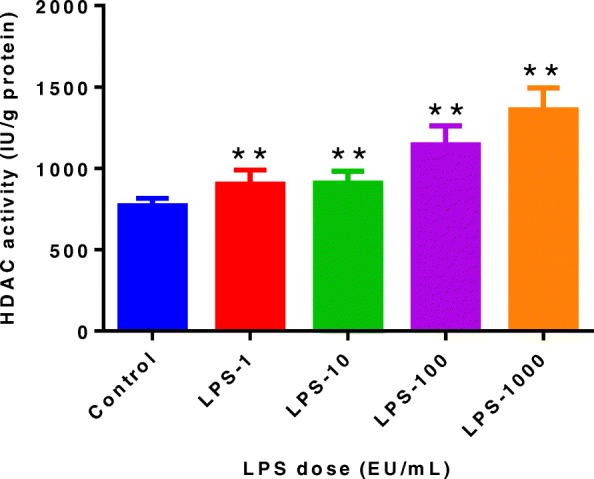
Effect of lipopolysaccharide (LPS) on histone deacetylase (HDAC) activity in MAC-T bovine mammary epithelial cells after treatment for 48 h with LPS at 0 (control), 1, 10, 100, and 1000 endotoxin units (EU)/mL, respectively. Data represent the mean and standard deviation (*n* = 8/treatment) and the asterisk indicates statistical difference between the indicated column and the control column (**, *P* < 0.01). The raw data were shown in Additional file 2: Table S2

To determine if LPS decreased histone H3 acetylation through increasing the activity of HDAC, we measured the activity of HDAC in MAC-T bovine mammary epithelial cells after LPS treatment. The activity of HDAC increased significantly (*P* < 0.01) after LPS treatment at different doses (Fig. 2), suggesting LPS can effectively enhance the activity of HDAC.

### Histone H3 acetylation levels after adding sodium butyrate

**Fig. 3:**
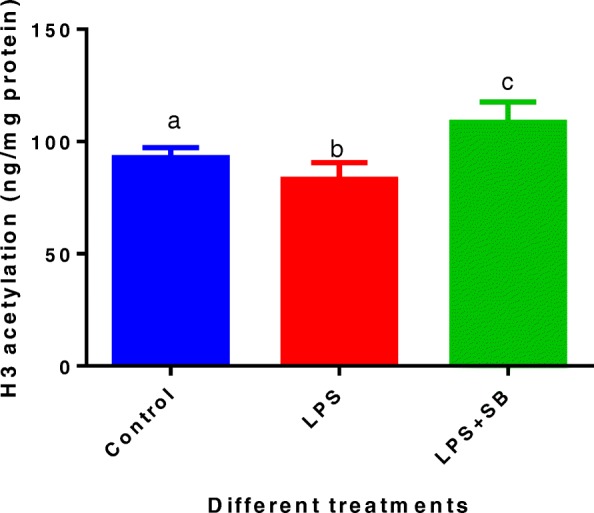
Acetylation of histone H3 in MAC-T bovine mammary epithelial cells among different treatments for 48 h: control, lipopolysaccharide (LPS, 100 EU/mL), and LPS (100 EU/mL) plus sodium butyrate (SB, 10 mmol/L). Data represent the mean and standard deviation (*n* = 6/treatment) and columns with different letters (a,b,c) indicate significant differences in histone H3 acetylation levels across treatments (*P* < 0.05). The raw data were shown in Additional file 3: Table S3

In order to determine whether SB, an HADC inhibitor, was able to counteract the adverse effect of LPS on histone H3 acetylation in MAC-T bovine mammary epithelial cells, the acetylation level of histone H3 after 48 h of treatment with LPS plus SB was determined (Fig. 3). H3 acetylation level of LPS treated cells was significantly lower than untreated control cells (*P* < 0.05). After adding SB into the LPS treatment, the H3 acetylation level was significantly higher (*P* < 0.05) than that of treatment with LPS alone or the control.

### mRNA expression of lactation-related genes

**Fig. 4:**
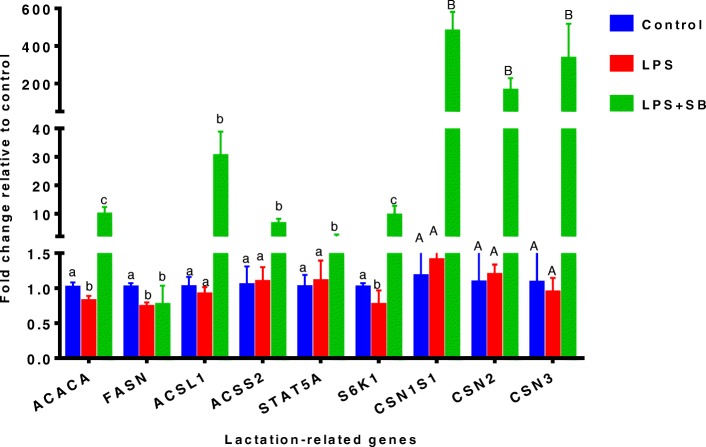
mRNA expression of lactation-related genes in MAC-T bovine mammary epithelial cells under different treatments for 48 h: control, lipopolysaccharide (LPS, 100 EU/mL), and LPS (100 EU/mL) plus sodium butyrate (SB, 10 mmol/L). Data represent the mean and standard deviation (*n* = 3/treatment). Columns with different lowercase letters (a,b,c) indicate significantly different values among treatments (*P* < 0.05), and columns with different uppercase letters (A,B) indicate highly significantly different values among treatments (*P* < 0.01). *ACACA*: acetyl-CoA carboxylase 1; *FASN*: fatty acid synthase; *ACSL1*: acyl-CoA synthetase long-chain family member 1; *ACSS2*: acyl-CoA synthetase short-chain family member 2; *STAT5A*: signal transducer and activator of transcription 5A; *S6K1*, ribosomal protein S6 kinase 1; *CSN1S1*: casein alpha s1; *CSN2*: casein beta; *CSN3*: casein kappa. The raw data were shown in Additional file 4: Table S4

To verify the correlation between histone acetylation and mRNA expression of lactation-related genes, we next determined whether SB was able to enhance mRNA expression of lactation-related genes through improving histone H3 acetylation in MAC-T bovine mammary epithelial cells exposed to LPS (Fig. 4). The results revealed that compared with control, LPS treatment significantly reduced the mRNA expression of *ACACA*, *FASN*, and ribosomal protein S6 kinase 1 (*S6K1*) genes (*P* < 0.05) in MAC-T bovine mammary epithelial cells, whereas the mRNA expression of acyl-CoA synthetase long-chain family member 1 (*ACSL1*), acyl-CoA synthetase short-chain family member 2 (*ACSS2*), signal transducer and activator of transcription 5A (*STAT5A*), casein alpha s1 (*CSN1S1*), casein beta (*CSN2*), and casein kappa (*CSN3*) was not affected by LPS (*P* > 0.05). Sodium butyrate was able to significantly suppress the adverse effects of LPS on mRNA expression of lactation-related genes, resulting in a tremendous increase in the mRNA expression of all genes except that the *FASN* mRNA expression was only numerically increased.

## Discussion

In the present study, we found that histone H3 acetylation in MAC-T bovine mammary epithelial cells decreased significantly after LPS treatment for 48 h at all the doses used in the experiment, although histone H4 acetylation was not significantly affected by LPS treatment. A study by Dong et al. showed when dairy cows were fed high-concentrate diets, the histone H3 acetylation level in the mammary tissue decreased [9]. More interestingly, their study found a negative relationship between LPS concentrations in the mammary blood and the histone H3 acetylation in the mammary tissue of dairy cows. Therefore, when bovine mammary epithelial cells are exposed to LPS, the LPS-induce histone H3 hypoacetylation may affect milk gene expression due to reduced transcription. The results of the present study confirmed that LPS treatment (100 EU/mL) significantly reduced the mRNA expression of lactation-related genes such as *ACACA*, *FASN* and *S6K1*.

On the contrary, Angrisano et al. found that histone H3 acetylation at interleukin- 8 (IL-8) gene promoter increased significantly after treating human intestinal epithelial cells with LPS for 30 min and 1 h [24]. Therefore, the effect of LPS on histone acetylation may display a selective pattern, depending upon the cell type and the primary function of the cell. In the case of intestinal epithelial cells or other immune cells, exposure to LPS will lead to inflammatory processes, and synthesis of inflammatory molecules such as IL-1, IL-6, and tumor necrosis factor-a (TNF-a) takes priority. Under the circumstance, a higher histone acetylation will facilitate the synthesis of inflammatory molecules.

The results of the present study showed that with increasing LPS doses, HDAC activity keeps rising. Therefore, the histone H3 hypoacetylation after treatment of MAC-T bovine mammary epithelial cells with LPS may be due to the rise of HDAC activity induced by LPS. There are few reports about the LPS-induced increase in HDAC activity in the mammary epithelial cells or tissue of dairy cows to date. However, similar studies have been reported in the literature. Aung et al. demonstrated that the mRNA expression of *HDAC*-4, 6, 7, 8 and 9 increased after LPS treatment of bone marrow-derived macrophages for 24 h [25]. Xing et al. found that after treating mouse lung fibroblasts (MIC-CELL-0040) with LPS for 72 h, the gene expression of *HDAC-4*, *HDAC-5* and *HDAC-7* was significantly increased [26]. Furthermore, in their study, LPS significantly increased the expression of HDAC-4, HDAC-5 and HDAC-7 proteins, and decreased the H3 and H4 acetylation levels [26].

Milk fat and milk protein are the most important milk components. The synthesis of milk fat and milk protein involves a number of genes. Acetyl-CoA carboxylase 1 (ACACA) is a key enzyme in the first step of fatty acid synthesis, this step is not a reversible reaction, and ACACA is the rate limiting enzyme of fatty acid synthesis. Fatty acid synthase (FASN) is an essential enzyme in fatty acid synthesis, and it catalyzes the synthesis of fatty acids from acetyl CoA and malonyl CoA through prolonging the short fatty acid carbon chain to a maximum of C16:0 [27]. The synthesized fatty acids will be activated by ACSL1 and enter the pathway of triglyceride synthesis. Our results showed that after LPS treatment, mRNA expression of milk fat genes such as *ACACA* and *FASN* was reduced. The study by Liu et al. showed that after LPS treatment of dairy cow mammary epithelial cells for 24 h, the gene expression of *ACACA*, cluster of differentiation 36 (*CD36*), fatty acid binding protein (*FABP*), *FASN*, peroxisome proliferator-activated receptor gamma (*PPARγ*), and sterol regulatory element binding protein 1 (*SREBP1*) involved in milk fat synthesis was significantly reduced compared with untreated cells [28].

Casein is the principal milk protein, accounting for 80% of the total milk protein. Casein consists of CSN1S1, CSN1S2, CSN2 and CSN3. The results of the present study showed that the mRNA expression of *CSN1S1*, *CSN2* and *CSN3* was not significantly affected by LPS. However, the mRNA expression of *S6K1* was significantly downregulated by LPS. Ribosomal protein S6 kinase 1 is a key protein translation regulator and is one of the most studied downstream molecules of the mammalian target of rapamycin (mTOR) pathway. The mTOR signaling pathway is not only a signal pathway for amino acid-driven protein synthesis, but also a key signal pathway for hormones such as growth hormone and insulin to regulate milk protein synthesis. The mTOR primarily regulates the translation of mRNA into protein through downstream S6K1. Valérie et al. demonstrated that the phosphorylation of mTOR increased 30 min after LPS treatment of the peripheral blood mononuclear cells, which enhanced the mTOR pathway activity [29]. In their study it was unknown whether the expression of *S6K1* was increased or decreased. In the present study, our results showed that after 48 h of LPS treatment, the expression of *S6K1* gene was significantly decreased, which may be one of the key factors for LPS-induced decrease of milk protein secretion. In addition, Janus kinase/Signal transducer and activator of transcription 5 (JAK/STAT5) is also an important signaling pathway involved in milk protein synthesis. During milk protein synthesis, some hormones and cytokines bind to their receptors and activate STAT5 (including STAT5A and STAT5B), and the activated STAT5 enters the nucleus to bind to target genes to regulate transcription [30]. Signal transducer and activator of transcription 5 can be used as a marker of milk protein gene transcription level [31]. However, there was no difference in *STAT5A* gene expression between LPS treatment and control in this present study.

In our present study, SB was able to suppress the adverse effects of LPS on the mRNA expression of all milk genes (*ACACA*, *ACSL1*, *ACSS2*; *STAT5A*; *S6K1*; *CSN1S1*, *CSN2* and *CSN3*) except that the *FASN* expression was only numerically increased after addition of SB into the LPS treatment. Therefore, SB, as an HDAC inhibitor, can significantly increase the lactation-related gene expression through counteracting the adverse effects of LPS on histone acetylation. Sodium butyrate appears to be likely to inhibit deacetylase activity due to its similarity to acetate. It may also be modified to butyryl-CoA, resulting in an increase in acetyl-CoA, which may alter acetylation levels through a mass action effect [32]. It is also worth mentioning that SB has proved very effective in reducing LPS-induced oxidative stress and apoptosis in cow mammary epithelial cells [33]. Sodium butyrate belongs to SCFAs, and because of its advantages in terms of high efficacy, low cost and high safety, it seems SB is a promising feed-in additive for improving lactation performance in dairy cows. More research is needed to validate and justify the use of SB for improving milk gene expression and for increasing lactation performance in cows exposed to LPS under various conditions such as high-concentrate feeding, poor hygienic environments, or/and mastitis and metritis in practical dairy production.

## Conclusions

In the present study involving bovine mammary epithelial cells, we demonstrated that there existed a linear relationship between LPS doses and the HDAC activity. Moreover, the histone H3 acetylation levels were reduced by LPS, while the histone H4 acetylation levels were not significantly affected by LPS. Therefore, endotoxin was able to induce histone H3 hypoacetylation possibly through effecting an increased HDAC activity. Sodium butyrate, as an inhibitor of HDAC, effectively suppressed the endotoxin-induced decline of histone H3 acetylation, resulting in improved mRNA expression of lactation-related genes in the bovine mammary epithelial cells.

## Methods

### Cell culture and treatments

The bovine mammary epithelial cell line (MAC-T cells) was used in this study and the cell line establishment methods were previously described by Huynh et al. [34]. The composition of the culture medium and the cell culture method were as described in our previous study [35]. Prior to the following experiments, the MAC-T cells were checked for their viability to ensure the growth curve of the cells was normal and satisfactory (Additional file 5: Figure S1).

In the first experiment, MAC-T cells were treated (*n* = 6) with LPS (*Escherichia coli* O111:B4; Sigma, L2630, USA) at doses of 0, 1, 10, 100, 1000 endotoxin units (EU)/mL (1 EU = 0.1 ng), respectively. The treatment was repeated with another batch of MAC-T cells. The nuclear protein was extracted after 48 h treatment, and the acetylation levels of histones H3 and H4 as well as the HDAC activity were measured. The doses of LPS were set to cover a wide range of plasma LPS concentrations in cows under different conditions that vary from healthy status through SARA and diseases. Whereas LPS might not be detected in the peripheral plasma of healthy cows, it was present in the mammary plasma with a level of about 1 EU/mL in cows fed high-concentrate diets [9] and with a level of up to 870 EU/mL in severe mastitis cows (our unpublished results).

In the second experiment, the treatments (*n* = 6) of MAC-T cells were as follows: control, LPS (100 EU/mL), and LPS (100 EU/mL) plus SB (10 mmol/L; MedChemExpress, HY-B0350A, USA). The treatment was repeated with another batch of MAC-T cells. After 48 h treatment, the nuclear protein and RNA were extracted and the acetylation levels of histones H3 and H4 as well as milk gene mRNA expressions were determined.

### Extraction of cell nuclear protein

After 48 h treatment, the cells were washed with DPBS (Solarbio, D1040–500, China) three times, digested with 0.25% trypsin/EDTA (Gibco, 25200056, USA), and centrifuged for 5 min at 3,000×g and room temperature to obtain clean cells. Histones were extracted with the cell nuclear protein extraction kit (Sangon Biotech Co., Ltd., C500009–0050, Shanghai, China). Briefly, cell membranes were lysed in a low osmotic and non-denatured system with protein phosphatases and protease inhibitors such as sodium pyrophosphate. Most of the cytoplasm protein and membrane protein were removed and the complete cell nucleus was extracted. Then the nucleus was decomposed using lysis buffer, and the cell nuclear proteins were extracted and purified. The BCA protein quantitative kit (Sangon Biotech Co., Ltd., C503021–0500, Shanghai, China) was then used to detect the extracted protein concentration in order to facilitate the subsequent accurate calculation.

### Assay for acetylation levels of histones H3 and H4 and activity of HDAC

The acetylation of histones H3 and H4 was determined by the bovine histone acetylation H3/H4 ELISA kit (Jiangsu Meibiao Biological Technology Co., Ltd., MB-5290A/MB-5291A, China). The bovine HDAC ELISA kit (Jiangsu Meibiao Biological Technology Co., Ltd., MB-4783A, China) was used to determine the HDAC activity. The level of acetylated histone H3 (AH-H3) was determined by the double antibody sandwich method. The microplates are coated with the AH-H3 antibody to prepare the solid-phase antibody. Samples were added into the microplates and then combined with the horseradish peroxidase (HRP) conjugated AH-H3 antibody to form the antibody-antigen-enzyme labeled antibody complex. After several washings, the substrate tetramethylbenzidine (TMB) was added to produce color. TMB was converted to blue color under the catalysis of HRP, and finally turned yellow under acidic conditions. The color intensity was positively correlated with the AH-H3 in the samples. The optical density (OD) was measured at 450 nm by a microplate reader (Bio-Rad, xMark™, USA). The concentration of AH-H3 was calculated as per the standard curve. The principle and procedures for histone H4 acetylation and HDAC activity assays were the same as those of histone H3 acetylation assay.

### RNA isolation and cDNA synthesis

The methodology used for RNA isolation and cDNA synthesis was described in detail in our previous study [35].

### Quantitative real-time PCR

Amplification and quantification of the prepared cDNA were as described in our previous study [35]. Gene-specific primer pairs were designed using Primer Premier 5.0 software for the genes including *CSN1S1*, *CSN2*, *CSN3*, *STAT5A*, *ACACA*, *FASN*, *S6K1*, *ACSL1*, and *ACSS2* (Table 1). The glyceraldehyde-3-phosphate dehydrogenase gene (*GAPDH*) was used as the internal control gene. Calculations of relative expression levels were performed using the 2^-ΔΔCT^ method [36] by taking into account the values of 3 replicates per treatment and at least 3 duplicates for each replicate.

**Table 1:** Primer sequences used for real-time quantitative PCR

Gene	Primer	Product size (bp)	GenBank accession No.
*CSN1S1*	F: CTTTTCAGACAATTCTACCAGCT R: AATTCACTTGACTCCTCACCAC	171	NM_181029.2
*CSN2*	F: AGTCCAAAGTCCTGCCTGTTCC R: TGCCATATTTCCAGTCGCAGTC	193	XM_015471671.2
*CSN3*	F: CAATACGCTGTGAGAAAGATGA R: AACTGGTTTCTGTTGGTAGTAA	122	NM_174294.2
*STAT5A*	F: CATGTCCCTCAAGAGGATCA R: TCATTGCTGCCAACACTG	108	NM_001012673.1
*ACACA*	F: GATCCAGGCCATGCTAAG R: CTGTTTCTCCAGCCACTC	103	XM_024979607.1
*FASN*	F: AGGACCTCGTGAAGGCTGTGA R: CCAAGGTCTGAAAGCGAGCTG	85	XM_005220997.3
*S6K1*	F: GGACATGGCAGGGGTGTTT R: GGTATTTGCTCCTGTTACTTTTCG	283	NM_205816.1
*ACSL1*	F: GCCGCATTTCACTTTTACTGC R: AGCTCTTTAGGGCAAACCCC	136	NM_001076085.1
*ACSS2*	F: GGGCGAATGCCTCTACTGC R: GCTGGGTGATGATGGATGG	254	NM_001105339.1
*GAPDH*	F: GGGTCATCATCTCTGCACCT R: GGTCATAAGTCCCTCCACGA	177	NM_001034034.2

*CSN1S1*: casein alpha s1; *CSN2*: casein beta; *CSN3*: casein kappa; *STAT5A*: signal transducer and activator of transcription 5A; *ACACA*: acetyl-CoA carboxylase 1; *FASN*: fatty acid synthase; *S6K1*: ribosomal protein S6 kinase 1; *ACSL1*: acyl-CoA synthetase long-chain family member 1; *ACSS2*: acyl-CoA synthetase short-chain family member 2; *GAPDH*: glyceraldehyde-3-phosphate dehydrogenase

### Statistical analyses

Statistical analyses were performed to compare the mean differences among treatments by using one-way ANOVA of SPSS (v.22). Mean differences for all variables were separated and compared using Duncan’s multiple comparison procedure. And data are presented as means ± standard deviation. Significance was declared at *P* < 0.05, and a tendency was considered if 0.05 < *P* < 0.10.

## Additional files

Additional file 1: **Table S1.** Authors’ original data for Fig. 1. (XLSX 11 kb)

Additional file 2: **Table S2.** Authors’ original data for Fig. 2. (XLSX 10 kb)

Additional file 3: **Table S3.** Authors’ original data for Fig. 3. (XLSX 9 kb)

Additional file 4: **Table S4.** Authors’ original data for Fig. 4. (XLSX 10 kb)

Additional file 5: **Figure S1.** Growth curve of the MAC-T bovine mammary epithelial cells. The growth of cells was measured by using CCK-8 (Cell Counting Kit 8, Dojindo, Japan). For each point of time, there were 12 wells in the cell culture plate (*n* = 12). The optical density (OD) was determined at 450 nm on a microplate reader (Bio-Rad, xMark™, USA). (PDF 6 kb)

## Data Availability

All data generated or analyzed during this study are included in this published article and its additional files.

## Abbreviations

ACACA: Acetyl-CoA carboxylase 1
ACSL1: Acyl-CoA synthetase long-chain family member 1
ACSS2: Acyl-CoA synthetase short-chain family member 2
AH-H3: Acetylated histone H3
CD36: Cluster of differentiation 36
CSN1S1: Casein alpha s1
CSN2: Casein beta
CSN3: Casein kappa
EU: Endotoxin unit
FABP: Fatty acid binding protein
FASN: Fatty acid synthase
GAPDH: Glyceraldehyde-3-phosphate dehydrogenase
HAT: Histone acetyltransferase
HDAC: Histone deacetylase
HRP: Horseradish peroxidase
IL: Interleukin
LPS: Lipopolysaccharide
mTOR: Mammalian target of rapamycin
PPARγ: Peroxisome proliferator-activated receptor gamma
S6K1: Ribosomal protein S6 kinase 1
SARA: Subacute ruminal acidosis
SB: Sodium butyrate
SCFA: short-chain fatty acid
SREBP: Sterol regulatory element binding protein 1
STAT: Signal transducer and activator of transcription
TMB: Tetramethylbenzidine
TNF: tumor necrosis factor
