# Supplementary figures and images for: Bacterial endotoxin decreased histone H3 acetylation of bovine mammary epithelial cells and the adverse effect was suppressed by sodium butyrate
Source: BMC Vet Res. 2019 Jul 29;15:267. doi: 10.1186/s12917-019-2007-5 (PMC6664593; doi:10.1186/s12917-019-2007-5)

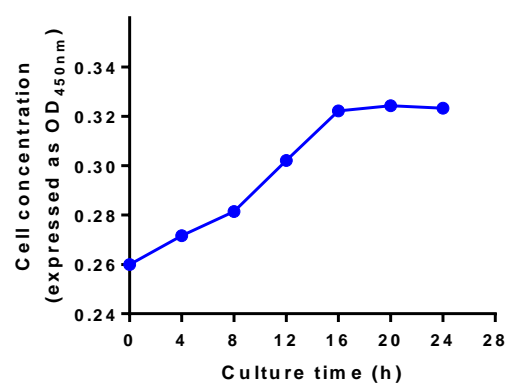

**Fig. S1**

Supplement: Supplementary file 5 — Figure S1. Growth curve of the MAC-T bovine mammary epithelial cells. The growth of cells was measured by using CCK-8 (Cell Counting Kit 8, Dojindo, Japan). For each point of time, there were 12 wells in the cell culture plate (n = 12). The optical density (OD) was determined at 450 nm on a microplate reader (Bio-Rad, xMark™, USA). (PDF 6 kb) [file 12917_2019_2007_MOESM5_ESM.pdf]
